# Supplementary material for: Cardiomyocyte-specific disruption of the circadian BMAL1–REV-ERBα/β regulatory network impacts distinct miRNA species in the murine heart
Source: Commun Biol. 2023 Nov 11;6:1149. doi: 10.1038/s42003-023-05537-z (PMC10640639; doi:10.1038/s42003-023-05537-z)
Supplement: Supplementary file 2 — Supplemental Material [file 42003_2023_5537_MOESM2_ESM.pdf]

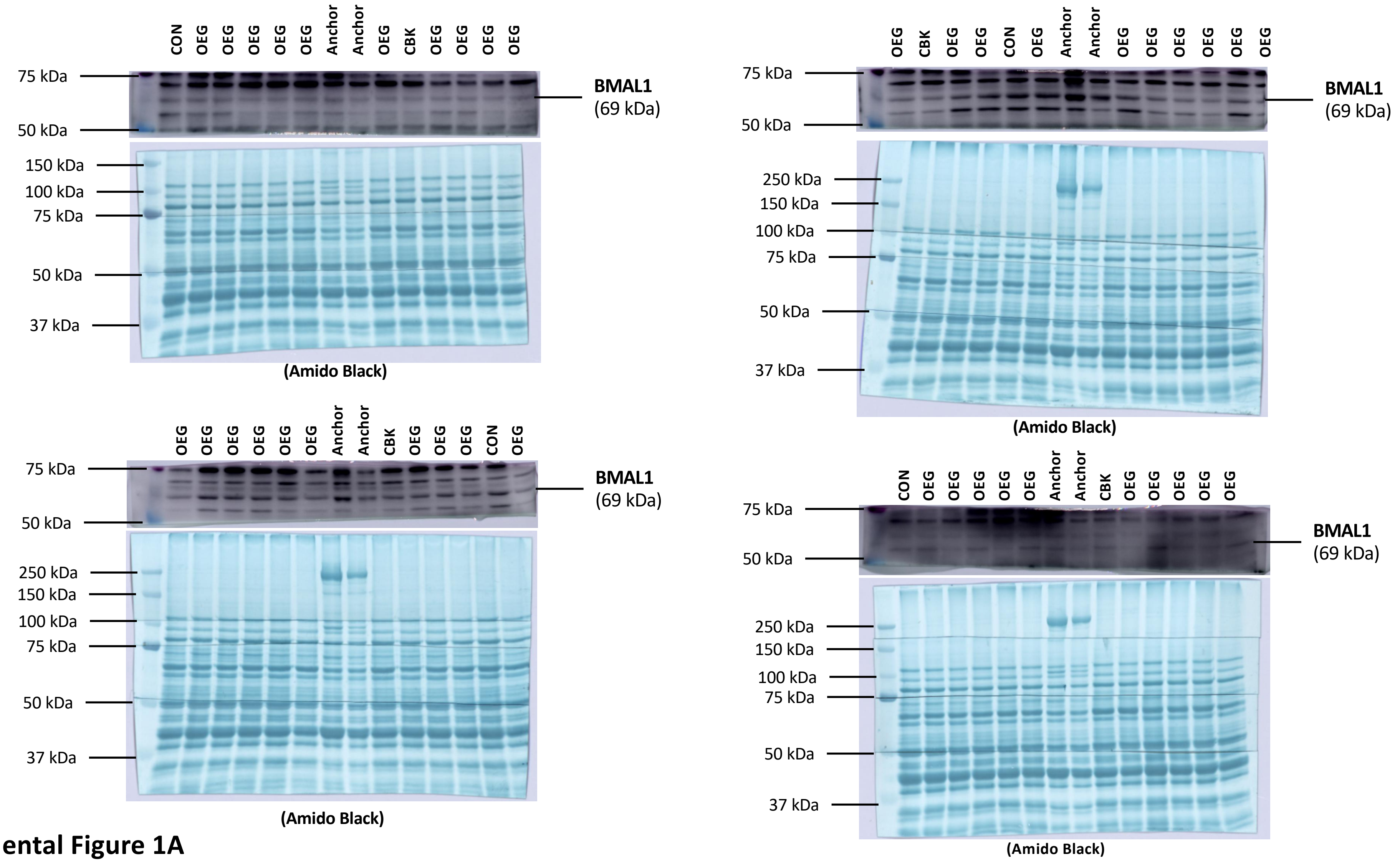

Supplemental Figure 1A

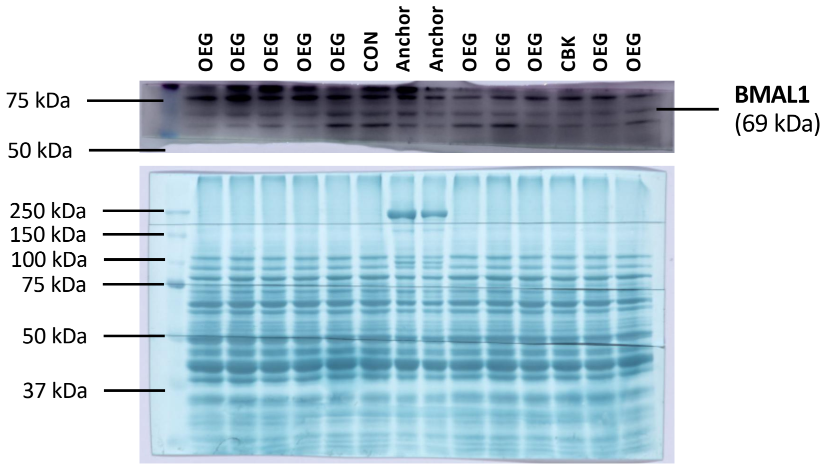

(Amido Black)

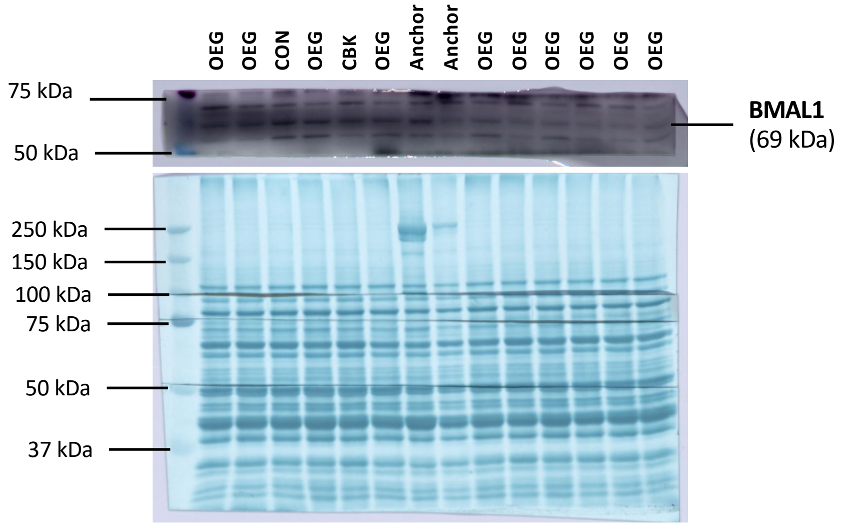

(Amido Black)

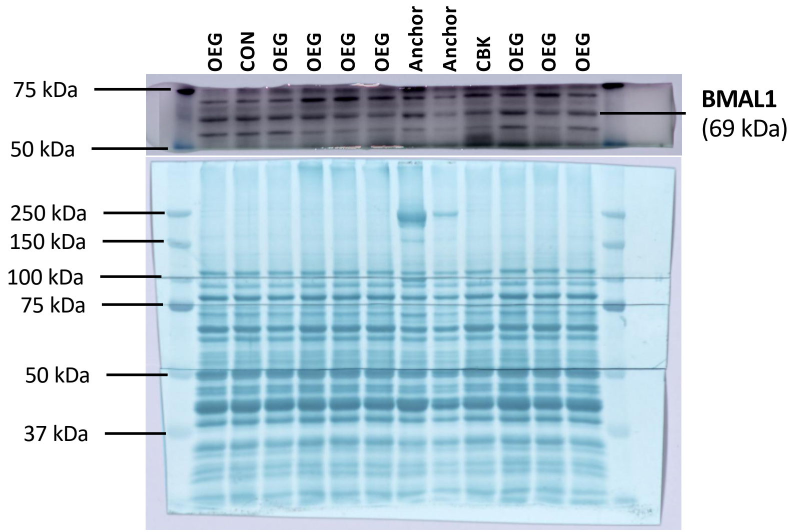

(Amido Black)

Supplemental Figure 1A

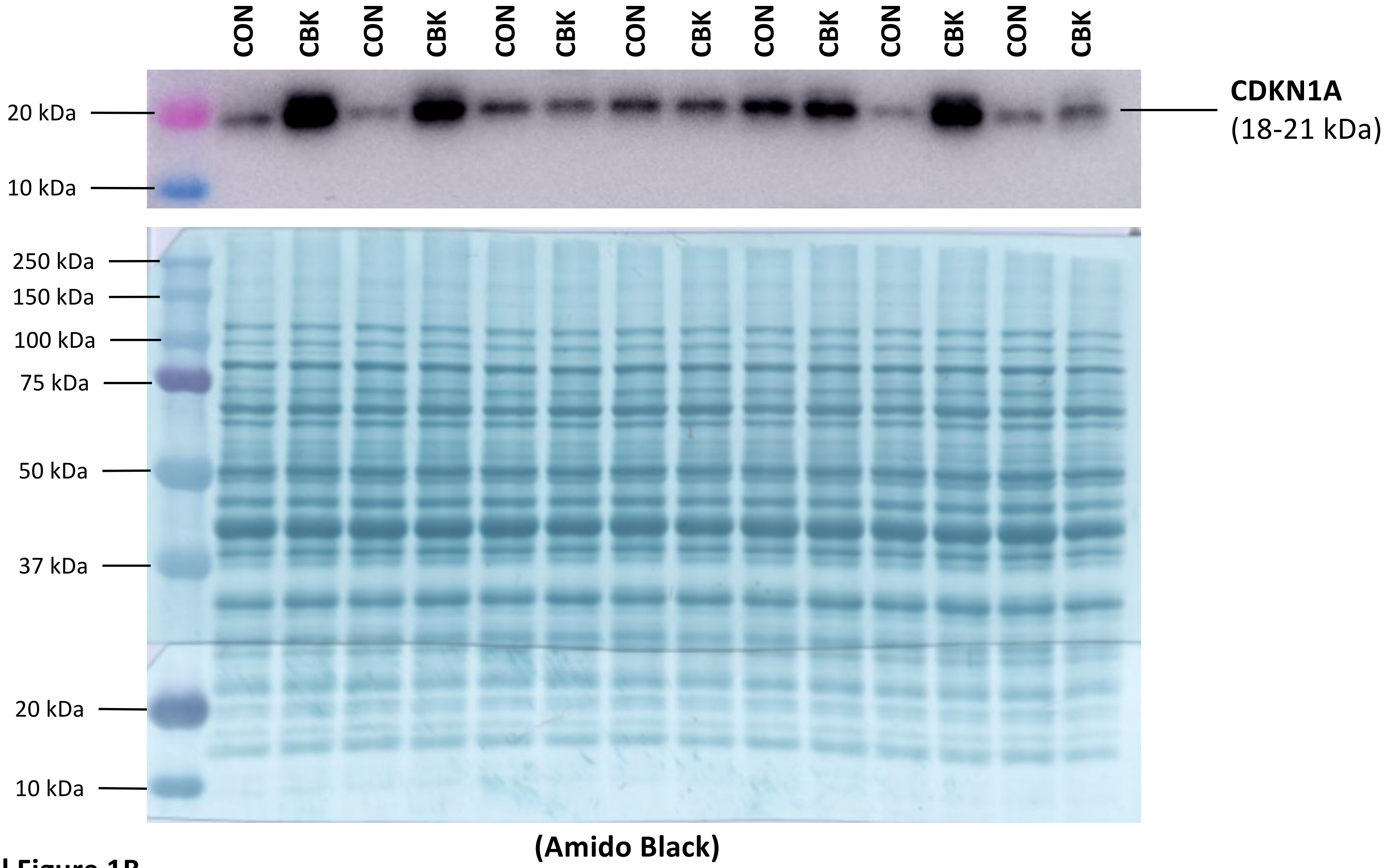

Supplemental Figure 1B

Supplemental Figure 1. Original Western Blot images utilized for data presented. Hearts were isolated from CBK and littermate CON hearts, followed by Western Blotting for BMAL1 (A) or CDKN1A (B). Original, uncropped images are presented here. OEG represents 'Other Experimental Group' (not reported).

		MESOR		Amplitude		Acrophase	
Figure	Target	CON	CBK	CON	CBK	CON	CBK
1Bi	*cry1*	1.70 ± 0.06	2.98 ± 0.09*	0.72 ± 0.08	0.59 ± 0.13	19.59 ± 0.44	20.52 ± 0.81
1Bii	*cry2*	1.47 ± 0.04	1.64 ± 0.05*	0.44 ± 0.06	0.17 ± 0.07*	12.03 ± 0.13	11.27 ± 0.38
1Biii	*per1*	3.45 ± 0.36	NR	3.12 ± 0.51	NR	10.75 ± 0.61	NR
1Biv	*per3*	2.68 ± 0.11	1.10 ± 0.07*	2.01 ± 0.15	0.89 ± 0.10*	12.56 ± 0.30	13.05 ± 0.44
1Ci	*hlf*	1.31 ± 0.08	0.68 ± 0.04*	0.52 ± 0.12	0.18 ± 0.06*	12.16 ± 0.89	14.10 ± 1.20
1Cii	*tef*	2.68 ± 0.11	NR	2.01 ± 0.15	NR	12.56 ± 0.30	NR
4Ai	miR-1a-2-5p	NR	0.97 ± 0.06	NR	0.23 ± 0.09	NR	22.80 ± 1.39
4Aii	miR-499-3p	NR	NR	NR	NR	NR	NR
4Aiii	miR-181a-1-3p	NR	NR	NR	NR	NR	NR
4Aiv	let-7c-1-3p	NR	NR	NR	NR	NR	NR
4Av	miR-23b-5p	NR	NR	NR	NR	NR	NR
4Avi	miR-31-5p	NR	NR	NR	NR	NR	NR
4Avii	miR-34a-5p	NR	NR	NR	NR	NR	NR
4Aviii	miR-215-5p	1.15 ± 0.04	NR	0.19 ± 0.05	NR	8.25 ± 1.03	NR
4Aix	miR-741-3p	NR	2.17 ± 0.08	NR	0.32 ± 0.11	NR	23.28 ± 1.28
4Bi	*cdkn1a*	2.09 ± 0.12	13.44 ± 0.58*	1.24 ± 0.17	4.34 ± 0.82*	22.21 ± 0.50	16.99 ± 0.73*
4Bii	*n5te*	NR	NR	NR	NR	NR	NR
4Biii	*npas2*	4.67 ± 0.43	NR	4.80 ± 0.60	NR	1.55 ± 0.49	NR
4Biv	*armc2*	1.33 ± 0.05	NR	0.17 ± 0.06	NR	15.89 ± 1.44	NR
4Bv	*herpud1*	1.41 ± 0.08	NR	0.36 ± 0.11	NR	16.72 ± 0.30	NR
4Bvi	*mid1ip1*	2.17 ± 0.20	NR	1.15 ± 0.28	NR	13.95 ± 0.25	NR
4Bvii	*rhobtb1*	1.85 ± 0.06	0.45 ± 0.02*	1.01 ± 0.08	0.07±0.02*	14.65 ± 0.33	12.33 ± 1.16
4Bviii	*npc1*	NR	NR	NR	NR	NR	NR
4Bix	*mylk4*	1.39 ± 0.05	NR	0.34 ± 0.06	NR	14.95 ± 0.73	NR
5B	*nr1d2*	1.61 ± 0.11	0.49 ± 0.02*	0.70 ± 0.15	0.30 ± 0.04*	11.71 ± 0.85	11.01 ± 0.44
5Ei	*dgat2*	1.59 ± 0.07	NR	0.50 ± 0.09	NR	16.93 ± 0.70	NR
5Eii	*ptpn3*	1.33 ± 0.05	NR	0.22 ± 0.07	NR	16.76 ± 1.22	NR
5Eiii	*usp54*	1.55 ± 0.09	0.97 ± 0.06*	0.41 ± 0.13	0.24 ± 0.08	14.95 ± 1.22	4.98 ± 1.32*

Supplemental Table 1. Cosinor analysis for mRNA and miRNA species in hearts isolated from CBK and littermate flox control (CON) mice. Tissues were isolated from mice at distinct times of the day, followed by assessment of mRNA and miRNA species by RT-PCR. Data are reported as mean ± SEM, for 6-8 mice per experimental group. *, $p<0.05$ for CON versus CBK. NR; not rhythmic.

Small RNA Species	Class	Control Expression	CBK Expression	Fold Change	Adjusted p-value
45S	rRNA	591,713.53 ± 37,490.77	757,999.63 ± 32,015.31	1.281	3.62E-02
ENSMUST00000082406	tRNA	2,659.76 ± 137.60	2,013.12 ± 141.54	0.757	4.52E-02
ENSMUST00000082423	tRNA	2,088.78 ± 124.87	1,491.90 ± 68.20	0.714	4.09E-04
ENSMUST00000083184	otherRNA	180.50 ± 7.69	142.30 ± 7.36	0.788	1.20E-02
ENSMUST00000083192	otherRNA	477.77 ± 22.03	688.42 ± 44.52	1.441	2.64E-04
ENSMUST00000083844	otherRNA	87.65 ± 4.64	156.08 ± 7.64	1.781	3.44E-12
ENSMUST00000104003	otherRNA	328.23 ± 11.78	248.54 ± 11.80	0.757	1.71E-04
ENSMUST00000122695	otherRNA	33.93 ± 1.47	43.67 ± 1.75	1.287	1.84E-02
ENSMUST00000133630	otherRNA	204.56 ± 22.29	116.51 ± 11.92	0.570	1.12E-02
ENSMUST00000153212	otherRNA	28.07 ± 2.55	47.65 ± 3.51	1.698	5.29E-03
ENSMUST00000154384	otherRNA	109.28 ± 7.67	74.82 ± 5.84	0.685	1.88E-02
ENSMUST00000158917	otherRNA	73.77 ± 2.81	91.22 ± 2.34	1.236	8.80E-04
ENSMUST00000177220	otherRNA	15.54 ± 1.13	24.68 ± 2.15	1.588	9.60E-03
ENSMUST00000180991	otherRNA	1,831.04 ± 85.51	1,311.24 ± 65.03	0.716	1.18E-03
ENSMUST00000181906	otherRNA	24.27 ± 0.99	31.63 ± 1.83	1.303	4.63E-02
ENSMUST00000184587	otherRNA	48.07 ± 1.72	39.77 ± 1.51	0.827	4.59E-02
ENSMUST00000185162	otherRNA	76.74 ± 5.21	111.06 ± 8.44	1.447	1.46E-02
let-7c-1-3p	miRNA	312.38 ± 17.71	443.60 ± 23.19	1.420	6.79E-04
miR-1298-5p	miRNA	16.68 ± 3.19	55.79 ± 2.57	3.344	1.38E-08
miR-135a-5p	miRNA	1,408.40 ± 122.52	488.01 ± 51.89	0.347	3.40E-10
miR-139-3p	miRNA	101.85 ± 9.13	170.26 ± 17.77	1.672	4.67E-03
miR-141-3p	miRNA	264.54 ± 24.50	174.18 ± 13.40	0.658	1.87E-02
miR-155-5p	miRNA	7,465.01 ± 324.14	5,394.58 ± 250.27	0.723	6.79E-04
miR-181a-1-3p	miRNA	1,567.57 ± 54.59	1,042.48 ± 58.36	0.665	6.12E-08
miR-181b-2-3p	miRNA	51.16 ± 4.05	36.90 ± 2.85	0.721	4.63E-02
miR-181b-5p	miRNA	3,533.32 ± 248.30	2,468.51 ± 156.89	0.699	2.87E-03
miR-1968-3p	miRNA	21.88 ± 1.54	37.96 ± 1.94	1.735	1.36E-07
miR-1968-5p	miRNA	38.93 ± 3.37	53.95 ± 3.31	1.386	4.61E-02
miR-1983	miRNA	146.60 ± 14.20	355.24 ± 29.68	2.423	1.20E-12
miR-1a-2-5p	miRNA	189.88 ± 19.56	110.67 ± 11.57	0.583	1.29E-02
miR-1b-3p	miRNA	48.04 ± 3.86	30.80 ± 2.32	0.641	8.40E-03
miR-200c-3p	miRNA	208.34 ± 11.65	141.26 ± 8.55	0.678	1.49E-04
miR-215-5p	miRNA	207.27 ± 16.65	375.70 ± 25.37	1.813	9.70E-06
miR-21b	miRNA	151.95 ± 7.38	187.82 ± 8.97	1.236	4.63E-02
miR-23b-5p	miRNA	41.48 ± 3.56	59.36 ± 4.19	1.431	3.16E-02
miR-28a-3p	miRNA	1,624.18 ± 63.75	1,988.44 ± 99.42	1.224	3.16E-02
miR-3057-5p	miRNA	70.18 ± 3.30	93.78 ± 5.60	1.336	8.41E-03
miR-3058-5p	miRNA	45.57 ± 3.13	31.93 ± 2.45	0.701	2.20E-02
miR-3100-5p	miRNA	27.13 ± 1.49	37.68 ± 1.92	1.389	1.66E-03
miR-31-5p	miRNA	1,696.59 ± 82.65	2,455.65 ± 160.85	1.447	7.03E-04

Small RNA Species	Class	Control Expression	CBK Expression	Fold Change	Adjusted p-value
miR-34a-5p	miRNA	12,684.10 ± 787.89	19,995.12 ± 1,147.87	1.576	2.46E-05
miR-383-5p	miRNA	3.99 ± 1.03	34.15 ± 2.20	8.559	8.91E-14
miR-425-3p	miRNA	510.22 ± 17.75	622.19 ± 22.84	1.219	8.87E-03
miR-465a-3p/miR-465b-3p/miR-465c-3p	miRNA	15.22 ± 1.79	39.10 ± 2.12	2.568	6.14E-08
miR-465c-5p	miRNA	6.51 ± 1.18	19.53 ± 1.53	3.000	3.79E-04
miR-470-5p	miRNA	14.96 ± 1.81	29.44 ± 2.05	1.968	2.24E-04
miR-490-3p	miRNA	903.09 ± 46.82	1,255.09 ± 90.21	1.390	3.75E-03
miR-490-5p	miRNA	80.95 ± 5.62	120.00 ± 8.13	1.482	5.71E-03
miR-499-3p	miRNA	613.57 ± 56.67	358.85 ± 33.73	0.585	3.08E-03
miR-499-5p	miRNA	37,782.93 ± 3,290.69	23,508.22 ± 2,253.45	0.622	2.61E-02
miR-5123	miRNA	55.14 ± 4.14	86.21 ± 4.25	1.563	4.55E-05
miR-582-3p	miRNA	122.19 ± 17.83	185.81 ± 13.94	1.521	4.37E-02
miR-669p-3p	miRNA	23.29 ± 1.66	16.33 ± 1.19	0.701	4.63E-02
miR-671-5p	miRNA	435.20 ± 22.71	598.69 ± 35.93	1.376	7.37E-03
miR-6958-3p	miRNA	45.05 ± 2.33	64.58 ± 2.65	1.433	4.75E-05
miR-700-3p	miRNA	318.46 ± 18.32	410.06 ± 20.22	1.288	2.54E-02
miR-7068-3p	miRNA	77.42 ± 3.83	113.00 ± 7.52	1.460	2.90E-04
miR-741-3p	miRNA	23.50 ± 1.70	106.00 ± 5.37	4.511	4.91E-59
miR-743a-3p	miRNA	7.43 ± 1.37	33.06 ± 1.89	4.447	1.38E-08
miR-743b-3p	miRNA	3.40 ± 0.81	16.82 ± 1.39	4.942	3.68E-06
miR-760-3p	miRNA	26.69 ± 4.96	60.49 ± 7.52	2.266	1.80E-02
miR-7689-3p	miRNA	19.74 ± 1.34	37.96 ± 2.17	1.923	3.44E-10
miR-871-3p	miRNA	3.72 ± 1.30	17.15 ± 1.77	4.615	8.87E-03
miR-9769-3p	miRNA	24.70 ± 1.47	17.33 ± 1.29	0.702	1.29E-02
piR_000580/gb/DQ540862/Mus_musculus: 11:106317126:106317155:Plus	piRNA	534.99 ± 26.21	763.62 ± 54.25	1.427	7.03E-04
tRNA-Asp-GTC-1-5	tRNA	511.11 ± 34.35	665.62 ± 32.63	1.302	1.16E-02
tRNA-Asp-GTC-1-9	tRNA	555.37 ± 63.02	847.40 ± 73.61	1.526	2.36E-02
tRNA-Gly-CCC-2-1	tRNA	27.72 ± 2.27	100.58 ± 20.02	3.628	1.35E-12
tRNA-Gly-CCC-2-2	tRNA	96.84 ± 10.85	386.91 ± 77.00	3.995	1.92E-10
tRNA-Gly-GCC-1-1	tRNA	1,798.42 ± 159.00	2,716.44 ± 254.97	1.510	1.54E-02
tRNA-Val-AAC-2-2	tRNA	70.29 ± 7.51	174.11 ± 37.52	2.477	1.72E-04
tRNA-Val-AAC-4-1	tRNA	80.61 ± 9.21	376.73 ± 96.08	4.673	2.50E-10
tRNA-Val-CAC-7-1	tRNA	18.68 ± 3.35	59.03 ± 18.00	3.159	2.36E-02

Supplemental Table 2. Differentially expressed miRNA species in CBK hearts. Hearts were isolated from CBK and littermate CON hearts at 4 hour intervals over a 24hr period, followed by small RNAseq. Two-way ANOVAs were performed using DESeq2A, to identify differentially expressed miRNA species between CBK and CON hearts. All data are presented as mean ± SEM.

GeneID	Control Expression	CBK Expression	Fold Change	Adjusted p-value
1110002E22Rik	1,239.01 ± 44.77	1,868.04 ± 80.23	1.508	9.36E-08
1110008P14Rik	375.96 ± 36.99	724.02 ± 114.03	1.926	1.55E-03
1700120C14Rik	163.05 ± 13.49	352.89 ± 65.42	2.164	3.34E-04
2310022B05Rik	1,441.24 ± 91.49	3,423.55 ± 259.81	2.375	2.30E-14
4631405J19Rik	173.82 ± 20.62	420.89 ± 65.50	2.421	6.55E-05
4632428C04Rik	173.17 ± 21.73	105.28 ± 8.66	0.608	7.99E-03
A530016L24Rik	735.85 ± 30.95	483.11 ± 53.07	0.657	4.55E-03
Aagab	447.96 ± 16.90	703.76 ± 23.24	1.571	6.36E-10
Abca12	150.33 ± 11.67	57.75 ± 5.92	0.384	3.32E-10
Abcb9	310.25 ± 10.11	506.39 ± 27.93	1.632	3.13E-08
Abcc9	3,584.74 ± 137.00	2,114.15 ± 231.60	0.590	3.61E-04
Abhd18	981.98 ± 63.46	531.36 ± 34.18	0.541	2.39E-08
Abhd6	187.11 ± 5.47	127.88 ± 10.90	0.683	3.98E-03
Abra	870.31 ± 75.45	1,495.04 ± 107.17	1.718	4.96E-05
Acsl6	205.03 ± 13.92	93.71 ± 6.03	0.457	1.29E-10
Acss1	6,290.76 ± 311.82	4,321.20 ± 251.62	0.687	1.72E-04
Acta1	5,691.55 ± 811.79	14,969.27 ± 1,811.92	2.630	1.04E-06
Actr3b	809.53 ± 64.72	460.74 ± 11.13	0.569	9.39E-08
Acy1	343.38 ± 10.10	208.71 ± 18.25	0.608	1.49E-05
Adam19	201.90 ± 21.01	923.56 ± 99.75	4.574	1.92E-24
Adamts15	136.76 ± 12.76	238.84 ± 18.04	1.746	3.03E-04
Adamts7	557.51 ± 33.67	349.91 ± 21.32	0.628	2.33E-05
Adamtsl2	257.39 ± 23.56	543.54 ± 39.37	2.112	1.70E-08
Adamtsl4	1,269.60 ± 60.46	797.38 ± 62.11	0.628	5.72E-05
Adck5	130.75 ± 6.92	193.84 ± 12.43	1.482	2.11E-03
Adcy1	85.85 ± 10.88	157.73 ± 21.92	1.837	1.35E-02
Adhfe1	1,252.06 ± 64.75	833.71 ± 31.27	0.666	3.29E-06
Adprhl1	10,342.49 ± 718.21	19,078.44 ± 1,356.74	1.845	5.31E-08
Adrb1	423.95 ± 26.74	266.46 ± 35.66	0.629	2.15E-02
Agpat3	3,791.46 ± 124.26	5,830.65 ± 213.45	1.538	8.65E-11
Agt	87.85 ± 11.24	301.36 ± 24.05	3.431	2.90E-15
Agtr1a	681.37 ± 34.64	452.02 ± 43.61	0.663	4.22E-03
Akap17b	83.02 ± 8.77	170.14 ± 42.17	2.050	1.68E-02
Akt1	3,871.55 ± 224.78	2,090.60 ± 168.02	0.540	9.24E-08
Aldh2	7,150.89 ± 287.69	4,732.80 ± 226.08	0.662	4.95E-07
Aldh9a1	741.51 ± 67.17	394.52 ± 21.49	0.532	3.82E-07
Alox5	358.39 ± 33.47	121.21 ± 13.39	0.338	7.45E-13
Amd1	1,358.51 ± 88.36	929.50 ± 48.87	0.684	5.06E-04
Ammecr1	254.06 ± 8.58	396.80 ± 43.31	1.562	1.98E-03
Amy1	280.93 ± 20.58	107.20 ± 9.91	0.382	5.56E-13
Ankrd1	20,139.77 ± 2,537.17	59,424.64 ± 6,516.41	2.951	9.78E-11
Ano10	1,517.10 ± 113.78	599.92 ± 34.45	0.395	6.55E-20

GeneID	Control Expression	CBK Expression	Fold Change	Adjusted p-value
Apex2	173.02 ± 8.72	86.94 ± 11.58	0.503	2.66E-05
Apln	266.15 ± 25.60	136.26 ± 15.48	0.512	1.06E-04
Arhgap24	297.17 ± 15.37	526.70 ± 43.60	1.772	4.16E-06
Arhgap9	128.28 ± 17.52	72.26 ± 5.46	0.563	5.25E-03
Arhgef10l	1,102.84 ± 61.28	1,758.36 ± 149.36	1.594	1.85E-04
Arhgef19	914.37 ± 70.11	1,399.88 ± 51.93	1.531	4.82E-05
Arhgef37	86.18 ± 9.48	150.32 ± 14.46	1.744	4.75E-03
Arl16	94.29 ± 5.43	134.27 ± 9.94	1.424	2.46E-02
Armc2	741.92 ± 73.11	186.10 ± 13.23	0.251	2.53E-27
Arrdc2	478.69 ± 72.17	290.21 ± 21.66	0.606	2.34E-02
Arrdc3	494.40 ± 40.11	323.07 ± 36.50	0.653	2.02E-02
Art4	345.71 ± 21.88	241.90 ± 17.18	0.700	7.63E-03
Art5	607.27 ± 60.50	283.54 ± 29.57	0.467	1.32E-06
As3mt	1,218.75 ± 28.62	846.20 ± 31.69	0.694	2.63E-08
Asb14	1,738.82 ± 31.53	1,179.67 ± 67.41	0.678	1.19E-06
Atf5	1,147.87 ± 57.92	1,702.82 ± 178.46	1.483	1.07E-02
Atp6v0a1	1,660.36 ± 57.27	2,489.48 ± 87.34	1.499	2.61E-09
Atp6v0e2	276.73 ± 28.73	417.59 ± 41.02	1.509	2.82E-02
Atp8a1	497.35 ± 26.02	271.42 ± 21.70	0.546	2.30E-07
Atp8a2	113.35 ± 6.51	235.61 ± 20.22	2.079	3.96E-08
Atxn1	501.39 ± 24.83	319.19 ± 14.89	0.637	1.39E-06
Auts2	391.28 ± 24.12	835.46 ± 44.87	2.135	1.06E-14
B4galt6	461.52 ± 31.18	743.33 ± 81.17	1.611	1.65E-03
Baiap2	198.38 ± 25.17	542.36 ± 97.46	2.734	2.16E-06
Bambi	301.58 ± 19.01	179.33 ± 15.15	0.595	1.59E-04
Banp	194.53 ± 17.85	352.46 ± 36.54	1.812	2.58E-04
Bckdhb	1,289.16 ± 51.17	727.91 ± 30.37	0.565	1.21E-14
Bcl2	167.84 ± 15.68	307.13 ± 28.72	1.830	1.01E-04
Bcl2l11	234.35 ± 14.66	157.11 ± 17.01	0.670	3.44E-02
Bdh1	2,680.56 ± 567.66	499.32 ± 86.53	0.186	6.24E-08
Bdnf	168.22 ± 7.25	107.08 ± 6.51	0.637	8.44E-05
Bhlhb9	292.54 ± 20.04	170.27 ± 10.79	0.582	2.00E-05
Bhlhe40	726.91 ± 44.84	465.08 ± 49.19	0.640	4.10E-03
Bhlhe41	666.84 ± 64.66	383.73 ± 33.57	0.575	2.96E-04
Bri3bp	927.19 ± 20.66	1,370.72 ± 82.76	1.478	1.07E-05
C030037D09Rik	129.58 ± 9.71	194.33 ± 14.56	1.500	8.07E-03
C1qtnf4	127.76 ± 9.84	226.38 ± 51.32	1.772	4.46E-02
Cabcoco1	386.80 ± 5.76	171.07 ± 16.41	0.442	2.14E-13
Cacnb1	142.18 ± 13.22	213.96 ± 7.74	1.505	4.58E-03
Cad	323.87 ± 21.42	201.74 ± 20.54	0.623	1.84E-03
Cadm4	433.12 ± 26.73	284.27 ± 16.08	0.656	2.76E-04
Calr3	310.90 ± 21.66	450.78 ± 34.99	1.450	1.07E-02

GeneID	Control Expression	CBK Expression	Fold Change	Adjusted p-value
Camk2b	281.08 ± 26.15	423.03 ± 38.10	1.505	2.65E-02
Car11	133.37 ± 25.32	224.99 ± 20.19	1.687	4.34E-02
Carnmt1	521.22 ± 19.58	367.56 ± 15.37	0.705	4.87E-05
Casq1	265.44 ± 38.25	819.94 ± 66.86	3.089	1.89E-11
Casq2	16,197.81 ± 471.08	24,741.00 ± 805.16	1.527	2.05E-12
Cavin4	1,479.97 ± 79.08	2,282.62 ± 143.10	1.542	2.00E-05
Ccdc141	2,028.13 ± 87.50	1,354.14 ± 72.73	0.668	5.27E-06
Ccdc189	164.04 ± 17.79	302.71 ± 69.90	1.845	3.05E-02
Ccdc25	575.06 ± 24.77	907.95 ± 149.97	1.579	2.01E-02
Ccn2	1,460.01 ± 186.18	2,434.29 ± 301.82	1.667	1.99E-02
Ccn5	314.88 ± 26.10	586.12 ± 76.33	1.861	4.16E-04
Ccrl2	248.59 ± 14.62	120.62 ± 13.68	0.485	1.65E-06
Cdh4	129.11 ± 18.99	234.23 ± 18.02	1.814	5.81E-03
Cdkl2	113.71 ± 12.05	192.35 ± 15.93	1.692	2.87E-03
Cdkn1a	232.71 ± 39.08	3,292.78 ± 461.40	14.149	6.68E-45
Cecr2	165.58 ± 7.49	115.65 ± 7.17	0.698	3.99E-03
Cenpa	760.06 ± 64.94	1,441.13 ± 95.14	1.896	1.49E-07
Cenpf	637.87 ± 65.42	386.88 ± 65.03	0.607	3.75E-02
Cep128	200.35 ± 13.77	53.79 ± 6.70	0.268	1.66E-16
Ces1d	3,352.18 ± 247.34	186.97 ± 40.35	0.056	1.97E-60
Cfap36	366.90 ± 22.45	521.61 ± 12.49	1.422	1.73E-04
Chd6	1,174.50 ± 149.32	638.12 ± 62.32	0.543	1.03E-03
Chid1	404.56 ± 30.16	572.16 ± 59.57	1.414	4.52E-02
Ciart	472.45 ± 37.36	119.24 ± 11.56	0.252	1.82E-26
Cidea	594.19 ± 40.31	1,138.25 ± 135.94	1.916	1.71E-05
Cited4	709.28 ± 54.33	1,079.47 ± 82.24	1.522	2.55E-03
Ckap4	486.08 ± 32.00	1,192.54 ± 36.73	2.453	6.46E-26
Ckb	1,454.08 ± 81.45	3,271.17 ± 349.73	2.250	3.01E-11
Clasp1	3,753.24 ± 202.73	6,411.46 ± 538.32	1.708	1.06E-05
Clec16a	576.67 ± 39.35	874.29 ± 87.02	1.516	8.79E-03
Clock	423.57 ± 23.34	826.06 ± 38.11	1.950	1.18E-13
Clpx	2,133.62 ± 73.51	3,067.98 ± 193.79	1.438	2.76E-04
Clu	16,200.40 ± 1,237.39	25,603.26 ± 937.20	1.580	4.51E-06
Cntn2	181.33 ± 18.32	101.12 ± 11.77	0.558	2.50E-03
Cobll1	1,170.56 ± 77.99	686.56 ± 58.06	0.587	7.19E-05
Col4a1	4,934.38 ± 188.60	7,197.14 ± 965.74	1.459	4.68E-02
Col8a1	360.59 ± 36.03	565.38 ± 63.29	1.568	3.42E-02
Colq	149.97 ± 22.43	582.68 ± 18.89	3.885	6.07E-20
Comt	2,941.63 ± 156.23	4,637.30 ± 375.09	1.576	1.92E-04
Coq10b	858.69 ± 40.53	330.31 ± 18.86	0.385	2.24E-26
Cox19	1,098.60 ± 81.49	2,102.31 ± 232.62	1.914	5.11E-06
Cpeb3	1,425.09 ± 74.48	823.50 ± 53.08	0.578	9.36E-08

GeneID	Control Expression	CBK Expression	Fold Change	Adjusted p-value
Creb3	604.36 ± 15.76	861.73 ± 56.98	1.426	2.49E-04
Crocc	215.46 ± 17.52	349.27 ± 30.40	1.621	2.79E-03
Cry1	234.85 ± 7.57	474.37 ± 38.19	2.020	4.51E-10
Csf1	911.47 ± 56.68	600.31 ± 41.15	0.659	3.83E-04
Csrp3	29,093.30 ± 1,723.63	42,680.28 ± 3,795.57	1.467	4.12E-03
Ctnna3	244.30 ± 14.63	159.03 ± 9.25	0.651	3.63E-04
Ctnnal1	520.27 ± 48.24	272.55 ± 7.27	0.524	2.39E-08
Ctsc	678.26 ± 38.43	448.77 ± 31.40	0.662	6.62E-04
Cutc	336.06 ± 17.28	213.33 ± 15.12	0.635	1.14E-04
Cyb5r3	2,012.56 ± 73.06	3,063.48 ± 250.22	1.522	1.90E-04
Cyfip2	2,513.18 ± 208.34	1,754.75 ± 162.66	0.698	3.74E-02
Cyria	140.87 ± 6.00	203.73 ± 17.22	1.446	1.22E-02
D830039M14Rik	101.76 ± 7.31	175.50 ± 23.43	1.725	5.46E-03
Dbi	2,002.79 ± 90.12	3,621.02 ± 627.87	1.808	2.44E-03
Dbp	2,294.95 ± 243.94	643.35 ± 55.11	0.280	5.04E-22
Dbt	1,290.70 ± 36.93	834.63 ± 50.94	0.647	3.40E-07
Dcakd	933.22 ± 21.35	448.08 ± 16.43	0.480	1.69E-32
Ddah1	98.61 ± 2.99	305.84 ± 11.98	3.101	4.57E-41
Ddit4	897.48 ± 139.84	2,390.93 ± 324.34	2.664	2.51E-06
Dgat1	480.08 ± 15.70	728.06 ± 51.03	1.517	3.15E-05
Dgat2	7,637.00 ± 530.74	2,082.44 ± 119.42	0.273	2.03E-41
Dhx37	337.10 ± 12.60	531.09 ± 54.89	1.575	1.39E-03
Dixdc1	203.02 ± 22.77	87.78 ± 8.41	0.432	3.82E-06
Dmd	1,035.27 ± 67.20	549.81 ± 38.92	0.531	7.57E-09
Dpysl3	690.23 ± 23.72	1,307.92 ± 61.79	1.895	8.57E-17
Dusp10	156.29 ± 8.32	84.15 ± 10.18	0.538	1.96E-04
Dusp27	989.86 ± 38.00	1,475.60 ± 85.31	1.491	1.30E-05
Dut	518.64 ± 33.72	361.12 ± 27.80	0.696	8.07E-03
Dync1li1	980.83 ± 39.33	1,566.59 ± 93.78	1.597	3.04E-07
E230013L22Rik	157.74 ± 16.08	246.61 ± 25.95	1.563	2.21E-02
Ech1	32,934.90 ± 1,544.19	21,484.21 ± 2,549.10	0.652	1.15E-02
Efnb3	1,847.81 ± 91.18	733.75 ± 52.91	0.397	5.86E-21
Egflam	419.32 ± 25.16	180.98 ± 14.95	0.432	9.72E-13
Ehd4	7,995.65 ± 294.90	12,396.37 ± 671.51	1.550	9.36E-08
Emilin2	1,089.67 ± 106.72	680.78 ± 57.52	0.625	3.63E-03
Emp2	1,176.02 ± 63.83	1,676.26 ± 200.44	1.425	4.02E-02
Erc1	348.05 ± 32.12	230.16 ± 22.05	0.661	3.15E-02
Exoc2	279.59 ± 10.82	186.62 ± 17.47	0.667	6.45E-03
F3	410.36 ± 14.33	136.10 ± 8.51	0.332	5.86E-36
Faap20	192.54 ± 19.23	323.86 ± 54.22	1.682	2.89E-02
Fah	1,144.60 ± 46.99	644.42 ± 63.00	0.563	1.36E-06
Fam110a	99.59 ± 7.70	160.63 ± 15.80	1.613	3.60E-03

GeneID	Control Expression	CBK Expression	Fold Change	Adjusted p-value
Fam131a	663.55 ± 32.15	445.45 ± 12.49	0.671	1.14E-06
Fam168a	611.43 ± 29.74	896.85 ± 53.41	1.467	4.06E-04
Fam189a2	1,302.57 ± 46.48	1,854.63 ± 50.12	1.424	5.77E-08
Fam81a	197.71 ± 12.01	122.57 ± 6.63	0.620	4.93E-05
Fbln1	2,584.60 ± 167.19	1,824.81 ± 105.65	0.706	2.52E-03
Fbp2	776.93 ± 36.81	306.50 ± 43.77	0.394	8.04E-09
Fbxo40	4,226.72 ± 135.80	6,127.95 ± 229.38	1.450	4.69E-08
Fdft1	940.98 ± 25.54	555.25 ± 30.77	0.590	5.00E-11
Fgf16	402.05 ± 36.71	238.71 ± 14.59	0.594	1.21E-04
Fgf9	185.76 ± 11.84	112.67 ± 9.73	0.607	5.10E-04
Fkbp10	578.90 ± 35.39	331.61 ± 25.57	0.573	3.31E-06
Flnc	5,146.42 ± 274.44	7,951.05 ± 705.77	1.545	6.61E-04
Foxo3	1,458.14 ± 102.88	991.98 ± 25.86	0.680	9.37E-05
Frmd5	2,328.88 ± 263.47	3,490.77 ± 223.99	1.499	3.31E-02
G0s2	1,114.57 ± 97.87	750.07 ± 51.37	0.673	5.72E-03
Galc	154.31 ± 8.11	227.67 ± 19.10	1.475	7.63E-03
Galm	523.23 ± 17.31	354.70 ± 7.36	0.678	1.27E-08
Galnt17	144.51 ± 7.32	96.35 ± 3.59	0.667	4.53E-04
Galnt18	208.10 ± 22.01	336.24 ± 20.83	1.616	2.71E-03
Gck	587.72 ± 76.12	1,821.58 ± 112.44	3.099	7.80E-13
Gja3	404.90 ± 19.03	266.43 ± 29.05	0.658	7.67E-03
Gjc1	335.27 ± 11.95	233.92 ± 20.84	0.698	7.48E-03
Gm10643	117.00 ± 8.10	186.22 ± 15.13	1.592	2.12E-03
Gm10800	104.01 ± 25.13	379.73 ± 122.58	3.651	8.37E-03
Gm14267	79.55 ± 9.91	126.99 ± 14.17	1.596	4.95E-02
Gm15417	335.52 ± 27.65	547.04 ± 94.55	1.630	3.79E-02
Gm24474	180.15 ± 12.55	62.51 ± 14.23	0.347	1.95E-05
Gm26782	92.79 ± 8.15	190.77 ± 45.84	2.056	1.56E-02
Gm36827	646.80 ± 51.79	230.79 ± 67.48	0.357	8.84E-05
Gm39214	91.64 ± 8.91	151.46 ± 11.72	1.653	3.76E-03
Gm45819	192.31 ± 32.89	35.64 ± 5.53	0.185	6.70E-12
Gm50100	379.87 ± 27.33	129.71 ± 16.80	0.341	6.47E-13
Gm9899	438.09 ± 30.21	632.04 ± 17.23	1.443	3.07E-04
Gna12	3,206.56 ± 77.46	2,049.40 ± 59.91	0.639	6.12E-16
Gpam	4,330.77 ± 345.83	2,598.91 ± 59.73	0.600	3.23E-07
Gpat3	277.09 ± 12.15	142.55 ± 10.66	0.514	5.88E-10
Gpc4	286.58 ± 14.33	474.53 ± 31.57	1.656	6.31E-06
Gpcpd1	4,154.33 ± 356.26	1,804.15 ± 167.26	0.434	2.37E-10
Gpr137	608.67 ± 29.34	884.44 ± 43.13	1.453	8.08E-05
Gpr22	604.49 ± 53.46	287.43 ± 43.57	0.475	7.32E-05
Grhpr	997.92 ± 38.30	1,498.85 ± 134.91	1.502	7.74E-04
Grk5	602.28 ± 23.47	1,085.04 ± 76.11	1.802	1.01E-08

GeneID	Control Expression	CBK Expression	Fold Change	Adjusted p-value
Gsg1l	274.44 ± 19.79	432.93 ± 36.83	1.577	1.08E-03
Gstm7	910.13 ± 24.43	1,747.08 ± 137.41	1.920	2.45E-11
Gstt1	440.11 ± 38.83	288.78 ± 29.50	0.656	1.74E-02
Gzmm	172.52 ± 7.73	106.23 ± 11.77	0.616	1.83E-03
H19	696.38 ± 62.10	387.54 ± 44.61	0.556	1.41E-03
H1f2	1,786.10 ± 37.26	2,924.01 ± 97.54	1.637	6.45E-18
H2bc21	151.31 ± 7.28	101.63 ± 9.69	0.672	1.41E-02
H2bc6	89.82 ± 6.83	160.34 ± 6.11	1.785	1.22E-06
H4c8	224.70 ± 16.40	516.06 ± 134.00	2.297	1.78E-03
Haghl	221.87 ± 15.79	346.21 ± 21.17	1.560	2.62E-04
Haus8	353.79 ± 19.43	693.44 ± 55.33	1.960	1.94E-08
Hcn2	610.19 ± 44.37	2,127.26 ± 99.19	3.486	2.78E-40
Hcn4	204.04 ± 14.13	70.14 ± 7.48	0.344	1.84E-13
Hdac4	633.41 ± 15.87	330.72 ± 18.08	0.522	5.60E-17
Hectd2os	106.91 ± 8.96	202.62 ± 20.30	1.895	5.60E-05
Herc3	521.61 ± 39.46	962.07 ± 115.94	1.844	4.07E-04
Herpud1	4,789.05 ± 307.27	2,486.28 ± 148.28	0.519	7.17E-11
Hfe	896.06 ± 51.41	1,367.95 ± 26.12	1.527	8.63E-08
Hk2	6,257.12 ± 299.37	4,373.77 ± 273.20	0.699	3.05E-04
Hlf	833.46 ± 84.25	451.41 ± 49.37	0.542	3.70E-04
Hmga1	393.79 ± 16.31	579.45 ± 31.88	1.471	1.01E-04
Hmgn2	1,187.55 ± 31.35	782.02 ± 34.66	0.659	1.05E-08
Hmgn3	104.89 ± 10.49	157.85 ± 10.37	1.505	1.72E-02
Homer2	946.99 ± 29.31	616.30 ± 79.04	0.651	5.94E-03
Hook1	205.84 ± 10.98	139.74 ± 10.18	0.679	3.92E-03
Hr	431.89 ± 34.36	854.48 ± 81.13	1.978	1.62E-06
Hsp90aa1	2,209.56 ± 206.39	4,153.91 ± 815.49	1.880	5.68E-03
Hspb3	211.13 ± 11.29	416.64 ± 35.96	1.973	1.76E-08
Hsph1	737.63 ± 33.20	1,168.07 ± 79.05	1.584	6.47E-06
Htatip2	566.83 ± 49.17	847.63 ± 63.93	1.495	4.75E-03
Iah1	233.99 ± 12.95	434.83 ± 32.13	1.858	4.10E-08
Idh1	1,059.69 ± 29.09	735.14 ± 14.80	0.694	2.18E-10
Idh2	40,045.04 ± 593.48	19,647.46 ± 950.14	0.491	4.49E-29
Ier3	779.81 ± 31.49	331.60 ± 19.97	0.425	1.26E-22
Ifrd2	534.83 ± 19.43	348.04 ± 38.24	0.651	8.06E-03
Ift74	102.91 ± 7.69	150.12 ± 13.24	1.459	3.65E-02
Ift81	1,179.06 ± 59.21	753.20 ± 45.68	0.639	7.04E-06
Igf2bp2	41.10 ± 4.11	242.85 ± 13.51	5.909	1.90E-44
Igfbp5	1,932.33 ± 157.57	1,278.57 ± 128.01	0.662	1.85E-02
Iigp1	788.22 ± 65.46	391.22 ± 76.44	0.496	1.90E-03
Il10rb	1,556.89 ± 51.41	1,001.22 ± 44.81	0.643	3.08E-09
Il15	738.55 ± 51.80	357.05 ± 32.67	0.483	1.67E-08

GeneID	Control Expression	CBK Expression	Fold Change	Adjusted p-value
Inpp5j	581.22 ± 15.10	934.03 ± 78.60	1.607	1.15E-05
Intu	526.48 ± 37.13	365.65 ± 33.29	0.695	4.13E-02
Irx1	140.56 ± 10.68	63.85 ± 10.00	0.454	2.37E-04
Isyna1	498.34 ± 16.13	347.91 ± 26.98	0.698	1.59E-03
Jak1	2,144.72 ± 111.35	1,439.35 ± 110.50	0.671	1.02E-03
Jam2	2,482.50 ± 61.80	1,370.28 ± 73.30	0.552	1.94E-15
Jph1	323.88 ± 26.10	202.46 ± 24.95	0.625	2.20E-02
Jpt1	1,863.75 ± 162.48	4,236.88 ± 677.89	2.273	4.27E-06
Kbtbd12	184.29 ± 11.41	276.48 ± 12.14	1.500	2.49E-04
Kcne1	92.48 ± 6.30	318.13 ± 37.62	3.440	3.89E-18
Kcnip2	4,100.10 ± 137.52	1,799.00 ± 61.09	0.439	1.36E-40
Kcnv2	244.11 ± 16.62	68.30 ± 3.06	0.280	7.17E-30
Kctd1	175.83 ± 7.22	307.55 ± 17.83	1.749	3.62E-08
Klf15	1,167.20 ± 53.13	783.22 ± 47.85	0.671	7.39E-05
Klhdc7a	103.75 ± 7.12	284.03 ± 32.75	2.738	4.78E-11
Klhdc8a	549.63 ± 37.19	1,343.69 ± 53.34	2.445	1.62E-24
Klhl30	1,015.25 ± 60.95	1,824.36 ± 64.14	1.797	2.77E-12
Klhl31	2,020.39 ± 48.67	3,025.95 ± 73.84	1.498	1.84E-14
Klhl38	230.03 ± 19.58	106.11 ± 13.81	0.461	6.37E-06
Ky	404.93 ± 50.34	211.04 ± 28.40	0.521	4.12E-03
Kyat3	483.44 ± 18.31	297.26 ± 17.13	0.615	1.31E-07
L3hypdh	182.15 ± 14.61	122.40 ± 5.07	0.672	3.65E-03
Lama5	2,627.43 ± 192.56	1,509.24 ± 118.39	0.574	8.62E-06
Lamb3	563.46 ± 22.54	391.52 ± 46.11	0.695	3.02E-02
Ldlr	131.27 ± 10.57	73.21 ± 11.16	0.558	8.88E-03
Leo1	227.36 ± 13.67	472.17 ± 20.25	2.077	3.22E-15
Lgals1	3,723.71 ± 391.13	7,016.63 ± 1,353.34	1.884	8.42E-03
Lgals3	125.96 ± 14.02	278.53 ± 43.48	2.211	3.88E-04
Lgals4	2,159.04 ± 192.89	852.42 ± 123.07	0.395	4.26E-07
Lingo3	243.64 ± 26.69	731.87 ± 76.16	3.004	1.40E-11
Lmo2	401.08 ± 19.20	240.87 ± 20.74	0.601	4.16E-05
Lnx1	159.78 ± 8.49	327.88 ± 35.44	2.052	6.67E-07
Lonrf1	210.68 ± 23.47	76.57 ± 11.76	0.363	1.29E-06
Lrfn4	337.94 ± 15.84	235.73 ± 13.12	0.698	8.44E-04
Lrp11	55.70 ± 5.34	166.98 ± 23.22	2.998	4.89E-08
Lrrc10	2,577.14 ± 187.06	3,711.34 ± 259.96	1.440	6.39E-03
Lrrfip1	1,359.35 ± 43.38	2,105.36 ± 62.34	1.549	1.69E-12
Lsm11	116.82 ± 8.00	165.80 ± 6.37	1.419	7.29E-03
Lzic	392.62 ± 33.17	569.39 ± 34.71	1.450	1.09E-02
Mal	381.05 ± 23.16	744.77 ± 29.21	1.954	2.68E-14
Maob	1,118.65 ± 85.92	407.97 ± 9.35	0.365	1.39E-24
Map1a	130.60 ± 7.67	224.28 ± 30.47	1.717	6.48E-03

GeneID	Control Expression	CBK Expression	Fold Change	Adjusted p-value
Mapk10	339.77 ± 20.96	496.63 ± 35.33	1.462	4.31E-03
Mapre3	928.28 ± 33.40	1,416.90 ± 83.09	1.526	5.52E-06
Mapt	2,219.39 ± 191.13	4,176.29 ± 385.55	1.882	3.42E-06
Mark1	183.81 ± 15.58	89.72 ± 7.82	0.488	4.03E-06
Mb21d2	224.41 ± 8.80	151.13 ± 9.97	0.673	6.51E-04
Mcts2	149.63 ± 7.56	217.07 ± 23.45	1.451	4.32E-02
Mdp1	966.45 ± 40.90	1,427.44 ± 123.32	1.477	1.74E-03
Me2	135.73 ± 7.87	205.08 ± 18.41	1.511	9.04E-03
Med10	306.90 ± 13.66	496.57 ± 30.48	1.618	2.49E-06
Metrn	492.45 ± 55.19	1,199.55 ± 254.59	2.436	5.72E-05
Mgat3	89.84 ± 9.01	140.08 ± 15.22	1.559	4.90E-02
Mid1ip1	2,374.25 ± 125.52	509.40 ± 33.01	0.215	4.84E-65
Miga2	1,255.96 ± 44.89	1,840.90 ± 116.83	1.466	1.07E-04
Mir208b	584.12 ± 36.58	400.68 ± 33.50	0.686	6.42E-03
Mknk2	4,371.08 ± 252.04	2,949.44 ± 174.53	0.675	2.99E-04
Mlf1	4,085.30 ± 134.43	2,696.29 ± 156.03	0.660	1.11E-06
Mllt11	131.45 ± 10.02	234.92 ± 20.33	1.787	5.73E-05
Mmp23	242.29 ± 14.82	356.56 ± 39.15	1.472	2.24E-02
Mpc1-ps	635.95 ± 26.82	414.51 ± 40.32	0.652	1.36E-03
Mpeg1	128.44 ± 4.17	189.22 ± 21.77	1.473	2.67E-02
Mphosph6	154.64 ± 6.84	98.65 ± 5.05	0.638	7.19E-05
Mpped2	420.43 ± 15.07	250.22 ± 20.30	0.595	2.51E-06
Mreg	492.77 ± 36.03	199.97 ± 23.35	0.406	2.85E-09
Mrln	26.67 ± 5.29	210.14 ± 27.42	7.879	4.38E-21
Mrps23	3,427.82 ± 185.11	5,411.44 ± 567.46	1.579	9.52E-04
Msrb1	2,279.33 ± 153.83	3,706.95 ± 590.99	1.626	1.75E-02
mt-Nd1	582,329.23 ± 31,377.71	395,860.71 ± 39,009.12	0.680	9.53E-03
Mtdh	1,854.30 ± 90.86	2,809.28 ± 213.37	1.515	2.81E-04
Mthfd2	53.52 ± 7.11	185.26 ± 19.02	3.461	1.60E-11
Mthfr	178.18 ± 11.90	290.04 ± 32.45	1.628	4.23E-03
Mttp	109.04 ± 14.94	280.14 ± 31.34	2.569	9.29E-07
Mybpc2	390.85 ± 35.84	687.76 ± 82.43	1.760	1.02E-03
Mybpc3	69,282.78 ± 2,952.57	108,038.44 ± 3,684.29	1.559	4.40E-10
Myl12a	5,715.01 ± 328.95	8,356.81 ± 469.46	1.462	1.74E-04
Mylk4	2,831.70 ± 306.64	661.46 ± 107.25	0.234	2.59E-14
Myo5b	678.04 ± 25.95	1,471.52 ± 82.91	2.170	8.63E-20
Myot	746.35 ± 38.66	215.99 ± 24.45	0.289	3.70E-23
Nabp1	615.84 ± 31.33	1,020.07 ± 129.40	1.656	3.97E-03
Nampt	3,353.32 ± 236.18	849.96 ± 38.26	0.253	6.56E-48
Napepld	87.33 ± 8.41	168.12 ± 13.36	1.925	6.08E-05
Ncs1	204.83 ± 9.41	291.80 ± 12.72	1.425	4.25E-04
Ndnf	111.37 ± 9.78	255.23 ± 23.63	2.292	4.61E-08

GeneID	Control Expression	CBK Expression	Fold Change	Adjusted p-value
Ndrg4	5,793.09 ± 458.25	10,484.30 ± 1,027.53	1.810	7.48E-05
Nfatc2	385.84 ± 26.98	262.50 ± 19.32	0.680	6.81E-03
Nfil3	225.22 ± 16.88	808.11 ± 44.61	3.588	1.24E-35
Ninj1	1,084.60 ± 64.95	1,664.41 ± 182.37	1.535	5.68E-03
Nlk	272.62 ± 16.09	401.35 ± 11.34	1.472	8.17E-05
Nol3	930.21 ± 69.98	1,698.83 ± 257.07	1.826	7.84E-04
Npas2	34.32 ± 4.13	170.78 ± 18.37	4.977	6.87E-20
Npc1	429.17 ± 53.33	200.96 ± 30.82	0.468	9.76E-04
Nppb	1,769.39 ± 377.59	4,670.08 ± 531.99	2.639	5.94E-05
Nqo1	1,162.73 ± 20.68	1,669.22 ± 79.22	1.436	6.41E-07
Nr1d1	1,018.40 ± 131.84	546.47 ± 80.63	0.537	6.88E-03
Nr1d2	1,836.63 ± 89.81	570.26 ± 43.22	0.310	4.89E-32
Nr3c2	381.04 ± 23.63	197.33 ± 12.68	0.518	3.33E-09
Nrep	564.57 ± 35.79	352.23 ± 33.53	0.624	5.18E-04
Nt5c2	733.70 ± 28.51	1,060.43 ± 39.97	1.445	2.00E-06
Nt5dc2	409.99 ± 5.43	277.60 ± 12.98	0.677	3.82E-07
Nt5e	202.06 ± 14.92	732.83 ± 85.86	3.627	3.72E-18
Nt5m	413.11 ± 25.97	617.41 ± 49.25	1.495	2.82E-03
Ntng2	170.71 ± 12.02	391.53 ± 46.74	2.294	1.09E-07
Nudt4	6,219.95 ± 451.28	3,866.30 ± 182.52	0.622	9.25E-06
Nudt7	1,607.12 ± 85.53	1,134.79 ± 85.05	0.706	5.76E-03
Nup62	433.73 ± 15.59	626.56 ± 15.42	1.445	1.14E-07
Ogfrl1	807.57 ± 75.26	1,386.59 ± 174.44	1.717	2.64E-03
Oplah	1,448.52 ± 84.21	753.17 ± 27.63	0.520	2.30E-14
Otulin	507.88 ± 31.51	719.53 ± 40.17	1.417	2.66E-03
P2rx5	327.31 ± 23.90	481.48 ± 30.99	1.471	4.00E-03
P2ry1	447.52 ± 41.24	249.76 ± 10.75	0.558	7.67E-06
P3h4	939.87 ± 47.58	475.94 ± 29.28	0.506	2.49E-12
P4htm	378.96 ± 23.55	554.51 ± 50.16	1.463	8.26E-03
Pak6	332.83 ± 25.38	525.12 ± 28.08	1.578	1.78E-04
Parp3	946.84 ± 40.59	1,384.79 ± 74.48	1.463	5.95E-05
Parvb	1,715.16 ± 49.64	2,445.53 ± 108.79	1.426	1.83E-06
Pcolce	1,332.50 ± 78.76	1,996.80 ± 143.34	1.499	4.42E-04
Pcp4l1	5,121.14 ± 145.66	3,277.47 ± 202.42	0.640	1.97E-07
Pctp	105.59 ± 10.77	168.63 ± 15.44	1.597	1.36E-02
Pcx	797.90 ± 33.14	518.71 ± 28.83	0.650	3.91E-06
Pde7a	580.24 ± 15.75	304.03 ± 9.75	0.524	1.57E-22
Pde7b	300.35 ± 25.91	172.25 ± 16.74	0.574	5.34E-04
Pdlim3	483.27 ± 34.65	337.72 ± 23.03	0.699	7.05E-03
Pdzd3	139.68 ± 15.89	218.02 ± 28.21	1.561	4.82E-02
Pebp1	3,955.62 ± 255.71	5,720.46 ± 716.28	1.446	4.37E-02
Peli2	269.15 ± 13.58	176.41 ± 15.84	0.655	4.92E-03

GeneID	Control Expression	CBK Expression	Fold Change	Adjusted p-value
Peli2_1	269.15 ± 13.58	176.41 ± 15.84	0.655	4.92E-03
Penk	222.74 ± 17.95	149.63 ± 9.47	0.672	9.80E-03
Per1	1,484.17 ± 107.11	899.50 ± 81.36	0.606	2.74E-04
Per3	857.06 ± 60.89	412.18 ± 29.52	0.481	1.05E-09
Pfkfb1	474.21 ± 49.42	140.40 ± 14.48	0.296	3.83E-14
Pfkp	911.50 ± 51.12	2,704.41 ± 158.71	2.967	6.50E-33
Phc1	298.31 ± 14.57	174.56 ± 18.28	0.585	2.56E-04
Phc2	842.67 ± 49.82	1,517.49 ± 67.36	1.801	7.51E-11
Phka2	376.18 ± 9.12	556.92 ± 10.73	1.480	9.18E-11
Phlda3	293.61 ± 25.70	472.73 ± 35.85	1.610	1.03E-03
Pik3r1	1,949.14 ± 149.54	1,376.64 ± 65.02	0.706	4.56E-03
Pik3r3	189.92 ± 13.89	312.50 ± 24.07	1.645	5.29E-04
Pim3	1,985.95 ± 134.09	1,113.31 ± 81.87	0.561	7.49E-07
Pknox2	236.53 ± 13.16	128.37 ± 7.33	0.543	2.50E-08
Pla2g5	884.33 ± 16.36	1,506.95 ± 81.56	1.704	4.01E-13
Plcd3	246.77 ± 10.32	438.41 ± 36.80	1.777	3.82E-07
Plcl2	519.81 ± 38.50	257.57 ± 50.87	0.496	1.49E-03
Plekhh3	672.16 ± 21.93	471.99 ± 12.53	0.702	2.64E-07
Plekho1	1,089.99 ± 82.74	1,550.20 ± 107.53	1.422	9.03E-03
Plscr1	237.02 ± 16.73	143.10 ± 16.97	0.604	4.49E-03
Plscr2	424.06 ± 24.87	281.63 ± 15.68	0.664	1.77E-04
Pnkd	1,438.10 ± 54.06	2,119.29 ± 189.59	1.474	1.55E-03
Popdc2	11,077.82 ± 276.24	15,809.77 ± 862.71	1.427	8.09E-06
Ppfia4	84.68 ± 3.39	201.05 ± 14.97	2.374	7.04E-14
Ppm1e	110.04 ± 4.79	181.50 ± 16.37	1.649	6.64E-04
Ppp1r1b	170.70 ± 36.77	45.36 ± 8.28	0.266	2.61E-05
Ppp1r3a	1,647.14 ± 122.06	1,052.67 ± 53.85	0.639	7.51E-05
Ppp1r3c	1,740.86 ± 98.91	3,210.30 ± 251.28	1.844	8.91E-08
Ppp2r3a	4,618.51 ± 188.49	2,743.74 ± 168.21	0.594	1.49E-08
Prkab2	858.92 ± 52.41	1,576.15 ± 77.05	1.835	1.91E-10
Prkar1a	6,771.39 ± 211.12	11,948.66 ± 760.49	1.765	1.91E-11
Prkcd	256.73 ± 12.90	375.97 ± 6.20	1.464	4.72E-06
Prkce	1,061.40 ± 36.50	714.94 ± 91.82	0.674	1.89E-02
Prkg1	224.19 ± 15.95	500.40 ± 44.13	2.232	1.08E-09
Prlr	202.95 ± 34.12	62.68 ± 9.70	0.309	5.42E-07
Prmt2	249.43 ± 18.21	383.27 ± 28.61	1.537	1.79E-03
Proser2	156.94 ± 15.51	277.43 ± 23.77	1.768	2.89E-04
Prps2	195.54 ± 16.16	128.36 ± 7.60	0.656	6.03E-03
Prune2	2,129.98 ± 108.28	3,507.83 ± 155.83	1.647	2.41E-09
Psmd8	2,165.33 ± 88.84	3,137.19 ± 196.24	1.449	1.24E-04
Ptgds	3,963.49 ± 537.91	1,373.62 ± 213.21	0.347	1.27E-06
Ptpn3	1,196.30 ± 103.52	537.00 ± 48.39	0.449	7.79E-09

GeneID	Control Expression	CBK Expression	Fold Change	Adjusted p-value
Ptpn4	331.35 ± 22.41	571.57 ± 47.92	1.725	5.54E-05
Ptpre	76.40 ± 7.24	175.68 ± 19.56	2.300	2.17E-06
Ptprn	158.06 ± 20.24	369.31 ± 38.69	2.337	5.13E-07
Ptpru	127.47 ± 13.24	75.09 ± 7.65	0.589	7.72E-03
Pxdn	862.06 ± 44.27	602.83 ± 59.99	0.699	2.13E-02
Pxmp2	686.26 ± 21.01	480.69 ± 36.04	0.700	1.33E-03
Pygl	93.14 ± 6.54	213.46 ± 4.77	2.292	6.44E-17
Qsox1	686.77 ± 21.08	1,085.48 ± 72.69	1.581	3.05E-06
Rab30	144.70 ± 7.23	227.55 ± 18.27	1.573	9.30E-04
Rab31	285.56 ± 12.44	588.65 ± 37.96	2.061	8.85E-14
Rab40b	76.73 ± 6.30	146.35 ± 13.74	1.907	1.28E-04
Rad54l2	245.82 ± 16.31	169.29 ± 17.78	0.689	4.49E-02
Rasgef1b	144.73 ± 19.24	65.81 ± 7.34	0.455	1.85E-04
Rcan1	1,295.97 ± 66.96	4,302.20 ± 243.42	3.320	1.59E-41
Relt	53.20 ± 5.94	169.59 ± 11.56	3.188	1.36E-14
Resf1	552.82 ± 61.55	1,134.09 ± 195.26	2.051	2.20E-03
Ret	124.63 ± 7.40	84.96 ± 8.24	0.682	3.31E-02
Rftn2	159.76 ± 10.20	294.63 ± 13.31	1.844	2.60E-09
Rgma	713.48 ± 27.48	466.30 ± 46.36	0.654	3.32E-03
Rgs12	335.43 ± 16.59	502.98 ± 44.13	1.499	2.27E-03
Rgs6	380.83 ± 15.91	197.76 ± 14.94	0.519	6.41E-10
Rgs7bp	175.49 ± 6.57	87.93 ± 11.33	0.501	3.18E-06
Rhbdl3	349.07 ± 30.46	210.33 ± 7.85	0.603	5.39E-05
Rhd	260.51 ± 44.33	126.32 ± 30.44	0.485	3.56E-02
Rhobtb1	2,871.42 ± 233.83	633.63 ± 48.76	0.221	6.84E-39
Rhoc	1,132.74 ± 67.87	1,819.04 ± 130.42	1.606	2.35E-05
Rhod	80.04 ± 5.31	123.05 ± 12.64	1.537	2.08E-02
Rmc1	550.93 ± 30.20	351.08 ± 13.72	0.637	1.39E-06
Rnf115	404.03 ± 27.35	654.48 ± 60.12	1.620	8.95E-04
Rnf166	904.24 ± 34.46	611.16 ± 23.80	0.676	3.73E-07
Ro60	358.12 ± 13.68	520.89 ± 44.11	1.455	2.68E-03
Rom1	434.20 ± 24.99	992.90 ± 117.79	2.287	1.96E-09
Rorc	655.43 ± 26.48	1,553.90 ± 27.36	2.371	6.69E-48
Rps4l	475.33 ± 60.23	923.44 ± 124.97	1.943	9.07E-04
Rragd	1,570.87 ± 69.75	2,501.26 ± 169.14	1.592	3.42E-06
Rras2	683.13 ± 19.95	1,273.11 ± 64.88	1.864	3.89E-16
Rrp12	395.76 ± 31.50	615.24 ± 64.65	1.555	1.51E-02
Rtn4	1,349.94 ± 60.99	2,083.01 ± 183.03	1.543	4.47E-04
Rtn4r	173.86 ± 24.50	510.26 ± 21.88	2.935	2.64E-10
Scamp5	243.43 ± 11.84	436.69 ± 18.11	1.794	1.55E-11
Scd4	210.45 ± 13.39	422.11 ± 55.98	2.006	2.43E-05
Scn4a	562.74 ± 63.73	350.26 ± 44.68	0.622	3.85E-02

GeneID	Control Expression	CBK Expression	Fold Change	Adjusted p-value
Scube2	268.63 ± 9.83	552.78 ± 77.79	2.058	1.68E-06
Scx	115.84 ± 11.79	260.21 ± 22.61	2.246	1.01E-07
Sema6c	290.18 ± 45.56	165.79 ± 7.55	0.571	5.73E-03
Serpina3n	224.42 ± 16.79	341.49 ± 35.86	1.522	1.43E-02
Serpine2	558.48 ± 32.54	378.32 ± 12.76	0.677	5.71E-05
Sfrp5	214.50 ± 61.07	45.21 ± 5.22	0.211	7.14E-07
Sh2d4a	238.81 ± 12.29	349.00 ± 32.23	1.461	1.35E-02
Sh3bgr	5,528.93 ± 379.81	8,682.21 ± 1,331.29	1.570	3.41E-02
Sh3bp5	756.23 ± 25.60	1,454.98 ± 148.33	1.924	4.04E-07
Shisa4	124.78 ± 8.92	197.17 ± 10.52	1.580	3.37E-04
Shld1	756.20 ± 74.59	196.81 ± 24.34	0.260	2.60E-17
Siva1	389.74 ± 21.70	257.26 ± 38.67	0.660	4.06E-02
Slc16a10	431.61 ± 27.90	266.13 ± 18.09	0.617	5.95E-05
Slc16a6	161.93 ± 13.45	277.63 ± 9.17	1.715	1.04E-06
Slc17a7	641.00 ± 117.32	3,120.82 ± 340.97	4.869	1.23E-16
Slc22a1	69.43 ± 8.77	144.95 ± 34.48	2.088	2.84E-02
Slc22a5	485.60 ± 28.41	315.99 ± 27.83	0.651	1.80E-03
Slc25a33	339.40 ± 16.65	220.16 ± 26.01	0.649	6.39E-03
Slc25a34	3,558.55 ± 157.19	2,314.21 ± 173.23	0.650	4.22E-05
Slc25a42	1,356.89 ± 127.27	883.56 ± 79.38	0.651	1.20E-02
Slc27a4	568.42 ± 16.30	810.16 ± 77.98	1.425	6.43E-03
Slc2a12	120.66 ± 4.88	79.83 ± 6.39	0.662	3.45E-03
Slc35f6	1,030.08 ± 36.87	1,539.67 ± 50.01	1.495	4.43E-09
Slc38a3	2,046.52 ± 121.51	1,222.39 ± 81.05	0.597	1.20E-06
Slc40a1	447.34 ± 26.88	243.01 ± 24.10	0.543	7.29E-06
Slc41a3	756.96 ± 48.03	5,801.23 ± 286.29	7.664	7.45E-118
Slc43a2	316.58 ± 15.72	542.44 ± 37.67	1.713	5.78E-07
Slc46a3	285.56 ± 21.43	165.97 ± 9.79	0.581	7.67E-06
Slc47a1	1,024.28 ± 55.72	1,650.95 ± 127.57	1.612	8.56E-05
Slc5a6	277.12 ± 16.37	195.51 ± 16.66	0.706	2.20E-02
Slco5a1	225.05 ± 10.17	121.41 ± 5.74	0.540	2.71E-10
Slf1	505.78 ± 23.93	336.06 ± 23.03	0.664	2.35E-04
Smco1	337.77 ± 25.32	232.64 ± 13.19	0.689	6.32E-03
Smim3	254.06 ± 13.53	173.69 ± 13.62	0.684	5.45E-03
Smtnl2	391.97 ± 19.13	188.37 ± 11.57	0.481	1.84E-13
Snta1	1,829.72 ± 75.95	2,773.42 ± 229.52	1.516	3.03E-04
Sord	3,159.66 ± 132.08	1,968.82 ± 64.57	0.623	4.72E-11
Sorl1	201.04 ± 14.41	301.90 ± 29.62	1.502	1.56E-02
Spata6l	334.57 ± 26.50	200.44 ± 15.42	0.599	1.92E-04
Spc24	150.64 ± 12.34	63.01 ± 8.04	0.418	4.61E-07
Sprr1a	5.47 ± 1.37	462.45 ± 100.49	84.572	1.91E-27
Spsb4	95.79 ± 5.71	230.67 ± 21.37	2.408	2.89E-10

GeneID	Control Expression	CBK Expression	Fold Change	Adjusted p-value
Srxn1	450.12 ± 12.42	660.94 ± 34.05	1.468	4.08E-06
Ssbp2	508.12 ± 10.51	290.68 ± 17.21	0.572	2.06E-11
Stc2	370.26 ± 37.95	247.79 ± 13.51	0.669	1.29E-02
Steap3	861.30 ± 40.48	524.93 ± 38.39	0.609	3.52E-06
Stox2	272.23 ± 29.28	455.58 ± 40.96	1.674	1.59E-03
Sv2a	144.85 ± 7.99	92.01 ± 10.54	0.635	1.15E-02
Syne1	681.57 ± 35.24	981.04 ± 96.31	1.439	2.11E-02
Syngr1	1,201.53 ± 65.70	1,725.21 ± 186.97	1.436	2.08E-02
Tbc1d16	1,329.88 ± 99.91	900.11 ± 83.56	0.677	1.47E-02
Tbcel	523.98 ± 25.76	883.35 ± 39.38	1.686	1.65E-09
Tcap	52,180.00 ± 4,450.32	13,866.49 ± 3,355.51	0.266	6.36E-10
Tdrkh	83.35 ± 6.63	169.56 ± 9.97	2.034	3.42E-08
Tef	3,899.01 ± 129.25	2,011.93 ± 77.39	0.516	8.63E-24
Tesc	2,678.48 ± 134.61	1,734.22 ± 269.19	0.647	3.13E-02
Tlr4	87.18 ± 4.84	146.99 ± 9.27	1.686	4.27E-05
Tmem159	470.65 ± 11.03	224.39 ± 12.99	0.477	3.30E-20
Tmem171	40.77 ± 5.69	199.97 ± 19.63	4.905	3.98E-19
Tmem192	313.67 ± 23.28	478.60 ± 66.67	1.526	3.85E-02
Tmem254a	194.38 ± 11.60	278.96 ± 16.90	1.435	5.05E-03
Tmem41a	257.02 ± 15.27	364.92 ± 25.16	1.420	8.79E-03
Tmem47	322.04 ± 14.93	159.85 ± 13.16	0.496	9.80E-10
Tmem51	88.15 ± 6.48	145.97 ± 9.96	1.656	5.40E-04
Tmem53	266.12 ± 16.78	457.86 ± 57.13	1.720	1.13E-03
Tmem62	96.62 ± 5.27	137.41 ± 9.22	1.422	1.73E-02
Tmtc1	1,573.05 ± 144.29	870.21 ± 74.14	0.553	7.78E-05
Tnfaip2	559.20 ± 38.94	930.81 ± 65.62	1.665	3.28E-05
Tnfrsf12a	514.22 ± 78.26	1,422.19 ± 152.35	2.766	5.26E-07
Tnip1	767.62 ± 25.30	1,192.15 ± 60.82	1.553	8.39E-08
Togaram2	142.70 ± 9.57	63.51 ± 7.85	0.445	1.09E-06
Tprn	107.20 ± 8.95	156.68 ± 13.33	1.461	3.02E-02
Trib1	424.46 ± 15.10	277.81 ± 20.64	0.655	1.01E-04
Trip10	2,519.37 ± 56.13	1,342.78 ± 55.95	0.533	2.14E-23
Tspan17	300.56 ± 24.29	617.36 ± 54.58	2.054	5.13E-08
Tspan9	1,330.81 ± 49.02	2,102.09 ± 136.44	1.580	1.08E-06
Tspo	634.62 ± 46.68	999.95 ± 116.42	1.576	9.00E-03
Tst	370.68 ± 23.34	180.42 ± 31.69	0.487	2.76E-04
Ttc39c	104.91 ± 4.50	151.12 ± 4.11	1.440	5.93E-04
Ttll1	1,285.62 ± 54.80	830.98 ± 65.71	0.646	2.64E-04
Ttll7	149.00 ± 8.11	262.99 ± 16.41	1.765	4.68E-07
Tubb2a	502.82 ± 36.62	726.66 ± 66.55	1.445	1.93E-02
Twf2	3,557.04 ± 253.62	5,306.90 ± 327.59	1.492	5.70E-04
Ube2ql1	225.39 ± 9.70	381.78 ± 22.46	1.694	1.99E-07

GeneID	Control Expression	CBK Expression	Fold Change	Adjusted p-value
Ubtd1	335.09 ± 14.22	542.56 ± 40.62	1.619	1.53E-05
Uchl1	182.77 ± 19.90	357.30 ± 72.23	1.955	7.22E-03
Uck2	662.11 ± 36.94	1,142.64 ± 67.65	1.726	4.02E-08
Ucp2	3,288.90 ± 324.67	6,720.45 ± 1,208.53	2.043	6.79E-04
Ucp3	1,119.50 ± 208.42	665.13 ± 71.63	0.594	2.95E-02
Ung	432.95 ± 43.70	166.72 ± 24.44	0.385	5.01E-08
Usp16	536.69 ± 28.85	906.57 ± 70.33	1.689	4.72E-06
Usp2	5,080.05 ± 144.69	3,001.98 ± 366.24	0.591	2.00E-04
Usp20	1,064.97 ± 18.93	1,616.73 ± 65.95	1.518	1.28E-10
Usp54	422.30 ± 24.01	256.57 ± 19.76	0.608	6.27E-05
Vwa3a	146.83 ± 13.55	270.73 ± 25.14	1.844	1.28E-04
Wee1	381.43 ± 36.31	134.15 ± 13.73	0.352	1.17E-11
Wfikkn2	151.49 ± 7.21	100.16 ± 10.65	0.661	1.53E-02
Whrn	561.72 ± 30.40	334.64 ± 34.81	0.596	7.84E-04
Wif1	157.64 ± 28.56	74.40 ± 8.18	0.472	5.02E-03
Wipf3	989.83 ± 47.13	675.65 ± 34.99	0.683	6.15E-05
Wnk2	1,677.61 ± 131.94	757.12 ± 57.35	0.451	2.45E-11
Wnk4	538.61 ± 8.54	317.49 ± 26.36	0.589	1.50E-07
Xirp2	7,573.65 ± 265.30	11,521.34 ± 958.58	1.521	3.10E-04
Xirp2_1	7,573.65 ± 265.30	11,521.34 ± 958.58	1.521	3.10E-04
Ypel2	265.16 ± 9.04	942.69 ± 39.59	3.555	1.50E-67
Zdhhc14	176.51 ± 6.15	113.99 ± 4.28	0.646	7.95E-06
Zfp275	180.49 ± 11.68	101.97 ± 9.98	0.565	3.26E-04
Zfp462	82.05 ± 8.74	140.70 ± 5.18	1.715	5.57E-04
Zfp536	161.75 ± 13.68	107.23 ± 5.41	0.663	6.81E-03
Zfp612	478.77 ± 16.91	307.13 ± 27.76	0.641	3.22E-04
Zfp970	416.62 ± 58.70	251.76 ± 14.05	0.604	9.04E-03

Supplemental Table 3. Differentially expressed mRNA species in CBK hearts. Hearts were isolated from CBK and littermate CON hearts at 4 hour intervals over a 24hr period, followed by small RNAseq. Two-way ANOVAs were performed using DESeq2A, to identify differentially expressed mRNA species between CBK and CON hearts. Criteria for inclusion in this table included differentially expressed mRNA species in CBK hearts with a log2 fold change value of ±0.5, an adjusted p-value of less than 0.05, and an average expression of greater than 100 counts. All data are presented as mean ± SEM.

mRNA Target	miRNA species
1110002E22Rik	miR-1a-2-5p
1110008P14Rik	miR-1a-2-5p
2310022B05Rik	miR-1a-2-5p, miR-499-3p, miR-499-5p
Aagab	miR-1a-2-5p
Abcb9	miR-1a-2-5p
Acss1	let-7c-1-3p
Acta1	miR-1a-2-5p
Acy1	miR-34a-5p
Adam19	miR-181a-1-3p, miR-181b-5p, miR-1a-2-5p
Adck5	miR-1a-2-5p
Adcy1	miR-181a-1-3p, miR-181b-5p, miR-1a-2-5p
Adrb1	let-7c-1-3p
Agt	miR-1a-2-5p
Akap17b	miR-181a-1-3p, miR-1a-2-5p
Aldh2	miR-215-5p
Ammecr1	miR-181a-1-3p, miR-1a-2-5p
Ankrd1	miR-1a-2-5p
Ano10	let-7c-1-3p
Apex2	miR-31-5p
Apln	miR-31-5p
Arhgef10l	miR-1a-2-5p
Arhgef19	miR-1a-2-5p
Arhgef37	miR-1a-2-5p
Arl16	miR-1a-2-5p
Armc2	let-7c-1-3p, miR-741-3p
Arrdc3	miR-31-5p
Atp6v0a1	miR-1a-2-5p
Atp6v0e2	miR-1a-2-5p
Atp8a2	miR-1a-2-5p
Atxn1	let-7c-1-3p, miR-23b-5p, miR-31-5p, miR-34a-5p
Auts2	miR-499-3p, miR-499-5p
B4galt6	miR-181a-1-3p, miR-181b-5p, miR-1a-2-5p
Baiap2	miR-181a-1-3p, miR-1a-2-5p
Banp	miR-1a-2-5p
Bcl2	miR-181a-1-3p, miR-181b-5p, miR-1a-2-5p
Bcl2l11	let-7c-1-3p
Bdnf	miR-31-5p
Bhlhe40	let-7c-1-3p, miR-31-5p
Bhlhe41	let-7c-1-3p, miR-23b-5p
Bri3bp	miR-181a-1-3p, miR-181b-5p, miR-1a-2-5p, miR-200c-3p
Cacnb1	miR-1a-2-5p
Calr3	miR-1a-2-5p

mRNA Target	miRNA species
Car11	miR-1a-2-5p
Casq2	miR-1a-2-5p
Ccdc141	let-7c-1-3p, miR-34a-5p
Ccdc25	miR-181a-1-3p, miR-1a-2-5p
Cdkn1a	miR-1a-2-5p
Cecr2	let-7c-1-3p
Cenpa	miR-1a-2-5p
Chd6	miR-31-5p
Chid1	miR-1a-2-5p
Ckap4	miR-1a-2-5p
Ckb	miR-1a-2-5p
Clasp1	miR-181a-1-3p, miR-499-3p, miR-499-5p
Clock	miR-1a-2-5p
Clpx	miR-181a-1-3p, miR-1a-2-5p
Clu	miR-1a-2-5p
Col4a1	miR-181a-1-3p, miR-1a-2-5p
Col8a1	miR-1a-2-5p
Comt	miR-499-3p, miR-499-5p
Cox19	miR-1a-2-5p
Creb3	miR-1a-2-5p
Cry1	miR-1a-2-5p
Csrp3	miR-181a-1-3p
Ctsc	let-7c-1-3p
Cyfip2	miR-23b-5p
Dbt	miR-31-5p
Ddah1	miR-181a-1-3p, miR-181b-5p, miR-1a-2-5p
Ddit4	miR-181a-1-3p, miR-181b-5p, miR-1a-2-5p
Dgat1	miR-1a-2-5p
Dgat2	let-7c-1-3p
Dhx37	miR-1a-2-5p
Dmd	let-7c-1-3p, miR-31-5p
Dpysl3	miR-181a-1-3p, miR-181b-5p, miR-1a-2-5p
Dusp27	miR-1a-2-5p
Ehd4	miR-1a-2-5p
Emp2	miR-1a-2-5p
Erc1	let-7c-1-3p, miR-34a-5p
Fam168a	miR-181a-1-3p, miR-1a-2-5p
Flnc	miR-1a-2-5p
Foxo3	miR-31-5p
Frmd5	miR-1a-2-5p
Galc	miR-1a-2-5p, miR-499-3p, miR-499-5p
Galm	let-7c-1-3p, miR-215-5p

mRNA Target	miRNA species
Gpam	let-7c-1-3p
Gpc4	miR-1a-2-5p
Gpcpd1	let-7c-1-3p
Gpr22	let-7c-1-3p
Grhpr	miR-1a-2-5p
Grk5	miR-1a-2-5p
Gstt1	let-7c-1-3p
Haghl	miR-1a-2-5p
Haus8	miR-1a-2-5p
Herc3	miR-181a-1-3p, miR-1a-2-5p
Herpud1	miR-23b-5p
Hfe	miR-1a-2-5p
Hk2	miR-23b-5p, miR-31-5p
Hlf	let-7c-1-3p
Hmgn3	miR-1a-2-5p
Hook1	miR-31-5p
Hr	miR-1a-2-5p
Hsp90aa1	miR-141-3p
Htatip2	miR-1a-2-5p
Iah1	miR-1a-2-5p
Idh2	let-7c-1-3p
Ier3	let-7c-1-3p
Ifrd2	miR-34a-5p
Ift74	miR-1a-2-5p
Igfbp5	let-7c-1-3p
Jak1	let-7c-1-3p, miR-23b-5p, miR-31-5p
Jam2	let-7c-1-3p
Jph1	let-7c-1-3p, miR-31-5p
Klhdc8a	miR-1a-2-5p
Klhl30	miR-1a-2-5p
Klhl31	miR-1a-2-5p
Ldlr	let-7c-1-3p
Lrp11	miR-181a-1-3p, miR-1a-2-5p
Lrrfip1	miR-181a-1-3p, miR-1a-2-5p, miR-499-3p, miR-499-5p
Lsm11	miR-1a-2-5p
Lzic	miR-1a-2-5p
Map1a	miR-181a-1-3p, miR-181b-5p, miR-1a-2-5p
Mapk10	miR-181a-1-3p, miR-181b-5p, miR-1a-2-5p
Mapre3	miR-1a-2-5p
Mcts2	miR-1a-2-5p
Me2	miR-1a-2-5p
Med10	miR-1a-2-5p

mRNA Target	miRNA species
Metrn	miR-1a-2-5p
Mgat3	miR-181a-1-3p, miR-1a-2-5p, miR-200c-3p
Mid1ip1	miR-31-5p
Mknk2	let-7c-1-3p, miR-215-5p, miR-34a-5p
Mllt11	miR-1a-2-5p
Mmp23	miR-1a-2-5p
Mpeg1	miR-1a-2-5p
Msrb1	miR-1a-2-5p
Mthfd2	miR-1a-2-5p
Mthfr	miR-1a-2-5p
Mttp	miR-1a-2-5p
Mylk4	miR-741-3p
Myo5b	miR-1a-2-5p
Nabp1	miR-181a-1-3p, miR-181b-5p, miR-1a-2-5p
Napepld	miR-1a-2-5p
Ncs1	miR-1a-2-5p
Ndrg4	miR-181a-1-3p, miR-181b-5p, miR-1a-2-5p
Nfil3	miR-1a-2-5p
Ninj1	miR-1a-2-5p
Nlk	miR-1a-2-5p
Nol3	miR-1a-2-5p
Npas2	miR-181a-1-3p, miR-181b-5p, miR-1a-2-5p
Npc1	miR-215-5p
Nppb	miR-1a-2-5p
Nqo1	miR-1a-2-5p
Nr1d2	miR-31-5p
Nt5c2	miR-1a-2-5p
Nt5e	miR-181a-1-3p, miR-499-3p, miR-499-5p
Nt5m	miR-1a-2-5p
Nudt7	let-7c-1-3p
Nup62	miR-1a-2-5p
P2rx5	miR-1a-2-5p
P4htm	miR-1a-2-5p
Parvb	miR-1a-2-5p
Pcp4l1	let-7c-1-3p
Pctp	miR-1a-2-5p
Pcx	miR-215-5p, miR-31-5p
Pde7a	miR-23b-5p, miR-31-5p
Pdlim3	miR-34a-5p
Pebp1	miR-1a-2-5p
Penk	let-7c-1-3p
Per3	miR-31-5p

mRNA Target	miRNA species
Pfkfb1	let-7c-1-3p
Pfkp	miR-1a-2-5p
Phc2	miR-1a-2-5p
Phlda3	miR-1a-2-5p
Pik3r1	let-7c-1-3p, miR-31-5p
Pik3r3	miR-181a-1-3p, miR-181b-5p, miR-1a-2-5p
Plcd3	miR-1a-2-5p
Plcl2	let-7c-1-3p
Plekho1	miR-1a-2-5p
Ppfia4	miR-1a-2-5p
Ppm1e	miR-1a-2-5p
Ppp1r3c	miR-181a-1-3p, miR-181b-5p, miR-1a-2-5p
Ppp2r3a	miR-31-5p
Prkab2	miR-1a-2-5p
Prkcd	miR-181a-1-3p, miR-181b-5p
Prkce	miR-31-5p, miR-741-3p
Prkg1	miR-1a-2-5p
Prmt2	miR-1a-2-5p
Prune2	miR-1a-2-5p
Psmd8	miR-499-3p, miR-499-5p
Ptpn3	let-7c-1-3p, miR-31-5p
Ptpn4	miR-181a-1-3p, miR-1a-2-5p, miR-499-3p, miR-499-5p
Ptpre	miR-1a-2-5p
Ptprn	miR-181a-1-3p
Ptpru	let-7c-1-3p
Pxdn	let-7c-1-3p
Pxmp2	miR-215-5p
Rab30	miR-1a-2-5p
Rab31	miR-181a-1-3p, miR-1a-2-5p, miR-499-3p, miR-499-5p
Rad54l2	let-7c-1-3p, miR-31-5p, miR-34a-5p
Rcan1	miR-1a-2-5p
Relt	miR-1a-2-5p
Rftn2	miR-1a-2-5p
Rgs12	miR-1a-2-5p
Rgs7bp	let-7c-1-3p
Rhobtb1	miR-31-5p, miR-34a-5p
Rhod	miR-1a-2-5p
Rnf115	miR-1a-2-5p
Rom1	miR-1a-2-5p
Rragd	miR-1a-2-5p
Rras2	miR-181a-1-3p, miR-181b-5p, miR-1a-2-5p
Rrp12	miR-1a-2-5p

mRNA Target	miRNA species
Rtn4	miR-181a-1-3p, miR-1a-2-5p
Scamp5	miR-181b-5p, miR-1a-2-5p
Scd4	miR-1a-2-5p
Scn4a	miR-31-5p
Serpina3n	miR-1a-2-5p
Serpine2	miR-31-5p
Sh2d4a	miR-1a-2-5p, miR-200c-3p
Sh3bgr	miR-1a-2-5p
Sh3bp5	miR-181a-1-3p, miR-1a-2-5p
Shisa4	miR-1a-2-5p
Slc16a10	let-7c-1-3p
Slc16a6	miR-181a-1-3p, miR-181b-5p, miR-1a-2-5p
Slc17a7	miR-1a-2-5p
Slc25a42	miR-741-3p
Slc35f6	miR-1a-2-5p, miR-499-3p, miR-499-5p
Slc38a3	let-7c-1-3p, miR-215-5p
Slc40a1	let-7c-1-3p
Slc41a3	miR-1a-2-5p
Slc43a2	miR-1a-2-5p
Smco1	miR-139-3p, miR-34a-5p
Smim3	let-7c-1-3p
Snta1	miR-1a-2-5p
Sord	let-7c-1-3p
Spsb4	miR-1a-2-5p
Srxn1	miR-1a-2-5p
Ssbp2	let-7c-1-3p, miR-23b-5p
Stc2	miR-34a-5p
Sv2a	let-7c-1-3p
Syne1	miR-181a-1-3p, miR-181b-5p
Syngr1	miR-1a-2-5p
Tbcel	miR-1a-2-5p
Tlr4	miR-1a-2-5p
Tmem171	miR-1a-2-5p
Tmem41a	miR-1a-2-5p
Tmem47	miR-31-5p
Tmem62	miR-181a-1-3p, miR-181b-5p, miR-1a-2-5p
Tnfaip2	miR-1a-2-5p
Tprn	miR-1a-2-5p
Trib1	let-7c-1-3p, miR-31-5p
Tspan17	miR-1a-2-5p
Ttc39c	miR-1a-2-5p
Ttll1	miR-31-5p, miR-5123

mRNA Target	miRNA species
Ttll7	miR-181a-1-3p, miR-1a-2-5p
Twf2	miR-1a-2-5p
Ube2ql1	miR-181a-1-3p
Ubtd1	miR-1a-2-5p
Uchl1	miR-181b-5p
Uck2	miR-1a-2-5p
Ucp2	miR-1a-2-5p
Ung	miR-34a-5p
Usp16	miR-181a-1-3p
Usp20	miR-1a-2-5p
Usp54	let-7c-1-3p
Vwa3a	miR-1a-2-5p
Whrn	miR-34a-5p
Xirp2	miR-1a-2-5p
Ypel2	miR-1a-2-5p
Zfp462	miR-181a-1-3p, miR-181b-5p, miR-499-3p, miR-499-5p

Supplemental Table 4. Putative mRNA targets of differentially expressed miRNA species in CBK hearts. Utilizing the mirnet omics tool, differentially expressed miRNA species were paired with putative mRNA targets that are also differentially expressed in CBK hearts.
