# Supplementary material for: Cardiomyocyte-specific disruption of the circadian BMAL1–REV-ERBα/β regulatory network impacts distinct miRNA species in the murine heart
Source: Commun Biol. 2023 Nov 11;6:1149. doi: 10.1038/s42003-023-05537-z (PMC10640639; doi:10.1038/s42003-023-05537-z)
Supplement: Supplementary file 3 — Description of Additional Supplementary Files [file 42003_2023_5537_MOESM3_ESM.pdf]

# Description of Additional Supplementary Files

**File name:** Supplementary Data

**Description:** Numerical source data underlying graphs and plots in the manuscript.
